# Efficiency of the Polycross and Controlled Hybridization Methods in Sweetpotato Breeding in Uganda

**DOI:** 10.5539/jas.v11n17p123

**Published:** 2019-10-15

**Authors:** Reuben T. Ssali, Godfrey Sseruwu, Bernard Yada, Gorrettie Ssemakula, Charles Wasonga, Wolfgang J. Grüneberg, Raul Eyzaguirre, Jan W. Low, Robert O. M. Mwanga

**Affiliations:** 1 International Potato Center, Kumasi Ghana; 2 National Agriculture Research Organisation, Mukono Zonal and Agricultural Research and Development Institute, Mukono, Uganda; 3 National Agriculture Research Organisation, National Crops Resources Research Institute, Namulonge, Kampala, Uganda; 4 International Potato Center, Kampala, Uganda; 5 International Potato Center, Lima, Peru; 6 International Potato Center, Nairobi, Kenya

**Keywords:** selection index, sweetpotato virus disease, Alternaria blight and, sweetpotato weevil

## Abstract

Sweetpotato is an important crop in many parts of the world especially in developing countries. It is used for both human consumption as well as livestock feed. It is an important source of carbohydrates, vitamin C, fibre, iron, potassium, protein and β-carotene. Its production is, however, constrained by several biotic and abiotic factors, including pests and diseases, low soil fertility, drought, cold and salinity. Breeding is one of the ways to overcome some of these constraints and in sweetpotato the polycross or controlled cross methods can be used. To determine which of the two methods was more efficient, genotypes generated by both methods were evaluated over two seasons at Namulonge and Kachwekano. The type of cross (polycross or controlled) was significantly (P ≤ 0.05) different for storage root yield, response to sweetpotato virus disease, Alternaria blight, and harvest index (HI). The controlled cross families had a significantly higher mean HI of 43.2% than the polycross families with a mean HI of 31.8%. Therefore, controlled crosses could be deployed to systematically increase the HI in sweetpotato breeding populations. Significant (P ≤ 0.05) differences were observed among families for all traits. This stresses that the parents used in a cross are very important in generating genotypes with desired attributes. It was apparent that both the polycross and controlled crosses are good methods for generating new sweetpotato genotypes in a sweetpotato breeding program. Where aggregate performance was considered (selection index) the controlled crosses method produced more (75% of the top 20 desirable genotypes) than the polycross method across the two sites. However, the best three genotypes over the two sites were from the polycross family of Ejumula. Therefore, sweetpotato controlled crosses could be very useful for population improvement using recurrent selection while polycrosses could be suitable for variety development. Both hybridization methods require cautious selection of parents to match the breeding objectives.

## 1. Introduction

Sweetpotato is the seventh most important food crop in the world (Loebnstein, 2016; FAOSTAT, 2016). It is an important source of carbohydrates, vitamin C, fibre, iron, potassium and protein (Woolfe, 1992) and is an excellent novel source of natural health promoting compounds, such as β-carotene and anthocyanins (Bovel-Benjamin, 2007). β-carotene is the precursor for vitamin A mainly from orange-fleshed sweetpotato varieties (Woolfe, 1992; Yanggen & Nagujja, 2006). Despite the importance of sweetpotato, its production and productivity are constrained by several biotic and abiotic factors (Kapinga & Carey, 2003). The main biotic factors include, diseases mainly sweetpotato virus disease (SPVD) (Gibson et al., 1997; Karyeija et al., 1998; Byamukama et al., 2004; Clark et al., 2012), Alternaria blight (*Alternaria* spp.) (Osiru et al., 2007a; Osiru et al., 2007b; Osiru et al., 2008; Clark et al., 2009) and the the major pests are sweetpotato weevils (*Cylas* spp.) (Stathers et al., 2003). The abiotic constraints include low soil fertility, drought, limited range of processing and utilization options and post-harvest problems such as lack of storage facilities and cold, heat and salinity (Grüneberg et al., 2015).

Sweetpotato breeding is one of the ways to overcome some of these constraints. In sweetpotato breeding, two hybridisation methods are used, namely, the polycross or open pollination and the controlled cross method (Wilson et al., 1989). However, sweetpotato breeding has several limitations because sweetpotato is highly heterozygous, outcrossing, a hexaploid (2n = 6x = 90) with complex genetics and segregation patterns (Martin, 1982). Its breeding system has numerous self-compatibility, self-incompatibility, cross-compatibility and cross-incompatibility challenges and has shy flowering habits in some regions of the world (Martin, 1965; Wilson et al., 1989). With incompatibility and sterility in sweetpotato coupled with poor seed set, obtaining the required cross-combinations is usually very difficult. Therefore, controlled crossing can be avoided by exploiting random, open-pollination in a polycross design (Jones & Dukes, 1980; Stuber, 1980; Jones, 1986). A polycross is the natural inter-crossing of a group of plants in an isolated crossing block (Stuber, 1980; Nyquist & Santini, 2007). In this design, only the female parent of each family is known, and the progeny are half-sibs (Stuber, 1980). However, for genetic studies the controlled cross method is used where both the female and male parents are known (Wilson et al., 1989). Both the controlled and polycross methods generate viable botanical seed and this study wanted to determine which of the two methods is more efficient in generating genotypes with desired attributes under Ugandan conditions.

## 2. Method

Two parents, ‘Ejumula’ and ‘Wagabolige’ were used to generate seeds in an open pollinated, polycross design of 100 parents (Mwanga et al., 2008) and controlled cross seeds were generated from four parents ‘Ejumula’ and ‘Wagabolige’ as female parents and ‘New Kawogo’ and ‘NASPOT 1’ as the male parents. In the polycross, it is assumed that the confounding effect due to different parents in the open-pollinated cross is similar for all crosses. ‘Ejumula’ is a released landrace in Uganda with low levels of field resistance to the SPVD, Alternaria leaf petiole and stem blight (Alternaria blight) and the sweetpotato weevil; has high β-carotene (orange-fleshed) and high dry matter content (34%) (Mwanga et al., 2007). ‘Wagabolige’ is also a released Ugandan landrace. It has moderate to high field resistance to SPVD, moderately resistant to the sweetpotato weevil and has low field resistance to Alternaria blight; it is white-fleshed (with no β-carotene) and high dry matter content (33%) (Mwanga et al., 2001).

For the controlled crosses, ‘Ejumula’ was crossed with ‘New Kawogo’ (male) and ‘Wagabolige’ was crossed with ‘NASPOT 1’ (male). ‘New Kawogo’ is a released landrace in Uganda. It has high field resistance to SPVD, moderate resistance to sweetpotato weevil and low field resistance to Alternaria blight; it is white-fleshed (with no β-carotene) and has high dry matter content (Stevenson et al., 2009; Mwanga et al., 2001). ‘NASPOT 1’ is a bred and released cultivar from the Uganda Root Crops Program at the National Crops Resources Research Program (NaCRRI), Namulonge. It has moderate field resistance to SPVD, is susceptible to the sweetpotato weevil and Alternaria blight; it is cream-fleshed (low β-carotene) and has high dry matter content (33%) (Mwanga et al., 2003).

Field experiments were conducted for two consecutive seasons at two locations representing two of the major sweetpotato producing eco-geographic zones in Uganda, namely, (i) the tropical montane zone at the Kachwekano Zonal Agricultural Research and Development Institute (KAZARDI), with bimodal rainfall average of 1319 mm annually and high Alternaria blight pressure, (ii) the tropical rain forest zone, at the National Crops Resources Research Institute (NaCRRI), Namulonge, with bimodal rainfall average of 1270 mm annually and high SPVD pressure. The experimental sites also differed in soil type and pH. The trials were established at the two sites during the first rains in April/May 2013 (2013A) and during the second rains in September 2013 (2013B).

A total of 94 open pollinated progenies from the two families (20 ‘Ejumula’ and 74 ‘Wagabolige’) and 94 progenies from the two controlled cross families (52 from ‘Ejumula’ × ‘New Kawogo’ and 42 from ‘Wagabolige’ × ‘NASPOT 1’) were selected for the trial. An alpha-lattice design with one replication was used at both locations during the two seasons. The vines were planted on ridges spaced 1.0 m apart. Each experimental plot consisted of 1 row, containing 5 plants spaced 0.3 m apart. The trials were kept weed free, and no supplemental irrigation was done, and no fertilizers or pesticides were applied.

Number of vines established and the first SPVD and Alternaria blight rating was done 1 month after planting (MAP). The pre-harvest data for SPVD and Alternaria blight were also collected 1 month before harvesting. SPVD and Alternaria blight rating described by Grüneberg et al. (2019) using a scale of 1 to 9 by scoring the severity of damage using the disease symptoms on leaves and stems. For SPVD , 1 indicated no virus symptoms, 2 = unclear virus symptoms, 3 = clear virus symptoms < 5% of plants per plot, 4 = clear virus symptoms at 6 to 15% of plants per plot, 5 = clear virus symptoms at 16 to 33% of plants per plot, 6 = clear virus symptoms at 34 to 66% of plants per plot (more than 1/3, less than 2/3), 7 = clear virus symptoms at 67 to 99 % of plants per plot (2/3 to almost all), 8 = clear virus symptoms at all plants per plot (not stunted), 9 = severe virus symptoms in all plants per plot (stunted).

For Alternaria blight (AB), 1 indicated no symptoms, 2 = unclear symptoms, 3 = clear symptoms at < 5% per plot, 4 = clear symptoms at 6 to 15% of plants per plot, 5 = clear symptoms at 16 to 33% of plants per plot (less than 1/3), 6 = clear symptoms at 34 to 66% of plants per plot (more than 1/3, less than 2/3), 7 = clear symptoms at 67 to 99 % of plants per plot (2/3 to almost all), 8 = clear symptoms at all plants (not fully defoliated), 9 = severe symptoms at all plants per plot (fully defoliated).

Harvesting was done five MAP at Namulonge and seven MAP at Kachwekano (the delay at Kachwekano was due to the cool climate in the highlands which causes a slower growth rate for most crops). At harvesting, the number of vines harvested was recorded, vine weight, flesh color, root defects, weevil damage, and the total storage root yield (RYTHA) was recorded in kilograms per plot and converted to t ha^-1^, total biomass (BIOM) was obtained by adding the vine weight to the RYTHA per plot and converted to t ha^-1^ and the HI was obtained by dividing the RYTHA by the BIOM.

Scoring for weevil damage (WED) was done using a scale of 1-9; where 1 = no damage, 3 = minor, 5 = moderate, 7 = heavy and 9 = severe damage, with numbers in between representing intermediate ratings. Storage root flesh colour (RFC) was determined using CIP colour chart, with a range of 1 to 30, where 1 = white and 30 = dark orange. Thr RFC was the converted to the β-carotene equivalent content in mg/100 g.

Analysis of variance (ANOVA) for each of the observed seven traits was done using the general linear model procedure, PROC GLM (SAS 9.4, SAS Institute, Cary, North Carolina) in two steps. Data were first classified by location, season, family, and genotype within family to assess the difference among the families. The model was,

$$y_{ijklm}=\mu +1_{i}+s_{j}+f_{k}+g(f{)}_{1(k)}+l_{ij}+{fl}_{ijk}+{gls}_{1(k)i]}\;(1)$$

where, l_i_ is the location, s_j_ is the season, f_k_ is family, g_l(k)_ is genotype within family, ls_ij_ is the season by location interaction, fls_ijk_ is family by season by location interactions, g(f)ls_l(k)ij_ is a residual that contains the interaction between genotype within family by season by location, plus within-trial error. The population mean, family minimum and maximum were calculated and the least significant difference (LSD alpha = 0.05) was used for comparing means of the families. In the second step data were classified by location, season, crosstype (whether the genotypes were derived from a polycross or controlled cross) and family within crosstype, with only crosstype as the fixed factor to assess the difference between polycross and controlled cross. The model was,

$$y_{ijk}=\mu +l_{i}+s_{j}+c_{p}+f(c{)}_{k(p)}+g(f{)}_{1(k)}+{ls}_{ij}+{cls}_{pij}+f(c)l_{ijk}+{gls}_{1(k)i}\;(2)$$

where, l_i_ is the location, s_j_ is the season, c_p_ is the crosstype, f(c)_k(p)_ is the family, g(f)_l(k)_ is the genotype within family, ls_ij_ is the location by season interaction, cls_pij_ is the crosstype by location by season interaction, f(c)ls_ijk_ is the family by season interaction, and gls_l(k)ij_ is a residual that contains the genotype by location by season interaction and within-trial error.

A selection index (SI) was used for discriminating between genotypes with a good aggregate of the desired traits from those with a poor aggregate in each of the four families. The traits weighted in the SI were: RYTHA; Alternaria blight severity scores; SPVD severity scores and WED. Since the traits (variables) were measured in different units with large differences in their magnitude and variance, they were standardized to make them comparable. Standardization was done separately for the controlled and polycross progeny after which the phenotypic value (Pij) for each progeny was obtained. The standardization was done as follows (Steel & Torrie, 1960):

$$P_{ij}=\left(x_{ij}-m_{i}\right)/s_{i}\;(3)$$

where, P_ij_ = Standardized phenotypic value; x_ij_ = Observed value of the trait i measured on genotype j; m_i_ = Overall mean of trait i; and s_i_ = Standard deviation of trait i.

The standardised phenotypic values were used to compute the SI for each genotype according to a modified formula of Ceballos et al. (2004). The specific formula for the SI was:

SI=(RYTHA×W4)−(SPVD×W3)−(WED×W2)−(AB×W1)                   (4)

W1-W4 are weights assigned to a trait where W is a weighting from 1 to 4. The magnitude of the weight depended on its importance. Since the importance of the traits varies with location, separate SI were developed for NaCRRI and Kachwekano. At NaCRRI, SPVD is the most important constraint thus was assigned a greater weight while at Kachwekano Alternaria blight is the most important and was assigned a higher weight than SPVD. At NaCRRI, the weights were assigned as follows: RYTHA = 4, SPVD = 3, WED = 2, AB = 1. At Kachwekano the weights were assigned as follows: RYTHA = 4, AB = 3, SPVD = 1 and WED = 2. A negative sign in the SI formula indicates that the trait was undesirable and contributed negatively to the SI.

Variance components for quantitative traits were estimated using data classified by genotypes within family across seasons and locations. The model was,

$$y_{ijklm}=\mu +l_{i}+s_{j}+g_{k}+{gls}_{ijk}\;(5)$$

where, l_i_ is the location, s_j_ is the season g_k_ is genotype, gls_ijk_ contains genotype by season by location interaction and within-trial error. Also, the broad-sense heritability (h^2^) of observed traits was calculated using the formula below:

$$h^{2}=\frac{\delta_{G}^{2}}{\delta_{G}^{2}+\frac{\delta_{GE}^{2}}{e}+\frac{\delta_{err}^{2}}{ge}}\;(6)$$

where, $\delta_{G}^{2}$ is the variance component due to the genotype, $\delta_{GE}^{2}$ is the variance component due to the interaction of genotype by environment, e is the number of environments and g is the number of genotypes.

## 3. Results

Overall, progenies derived from polycross and controlled crosses evaluated at Namulonge and Kachwekano during the 2013A and 2013B seasons had an average storage root yield of 9.1 t/ha, average biomass of 24.7 t/ha, average HI of 39%, and average β-carotene content of 0.8 mg/100 g on a fresh weight basis and average SPVD, Alternaria blight and weevil damage scores of 2.2, 1.9 and 1.9, respectively (Table 1). The controlled cross family ‘Wagabolige × NASPOT 1’ had the highest storage root yield, 10.3 t/ha, while the polycross family of ‘Wagabolige (op)’ had the least storage root yield of 6.4 t/ha (Table 1). Two families ‘Ejumula (op)’ and ‘Ejumula × New Kawogo’ had the least weevil damage score of 1.7, while the controlled cross family of ‘Wagabolige × NASPOT 1’ had the highest weevil damage score of 2.0. The least β-carotene content of 0.0 mg/100 g, was observed in the controlled cross family of ‘Ejumula × New Kawogo’ and the highest β-carotene content of 1.1 mg/100 g in the polycross family of ‘Ejumula (op)’. Biomass yield was highest in the controlled cross family of ‘Wagabolige × NASPOT 1’ of 25.4 t/ha and lowest in the controlled cross family of ‘Ejumula × New Kawogo’ at 21.0 t/ha. HI was highest in the ‘Wagabolige × NASPOT 1’ family, 44.3%, and lowest in the polycross family of ‘Wagabolige (op)’ 29%.

**Table 1:** Family means for traits observed at Namulonge and Kachwekano over two seasons in 2013

Family	Storage root yield (t/ha)	SPVD	Alternaria	Weevil damage	Biomass (t/ha)	Harvest index	β-carotene content (mg/100 g FWB)
Ejumula op	6.6	2.4	1.7	1.7	22.8	34.3	1.1
Wagabolige op	6.4	2.4	1.7	1.8	21.9	29	0.2
Ejumula × New Kawogo	7.9	2	2.3	1.7	21	39.1	0
Wagabolige × NASPOT 1	10.3	2.2	2	2	25.4	44.3	0.7
Mean	9.1	2.2	1.9	1.9	24.7	39	0.8
LSD_(0.05)_	1.3	0.5	0.4	0.1	4.5	16	0.1

Individual genotypes, (genotype (family)), differed significantly in their mean storage root yields, weevil damage and β-carotene content (Table 2). Also, families, (family (cross type)), differed significantly in their mean storage root yields, biomass yield, HI, SPVD, Alternaria blight, weevil damage scores and β-carotene (Table 3). The effect of location on the performance of families derived by either polycross or controlled crosses was significant for all the traits except β-carotene content and the effect of season on the performance of families was significant for all traits accept Alternaria blight and β-carotene content (Table 3).

**Table 2:** Family analysis of variance mean squares for seven sweetpotato traits of progenies derived from controlled cross and polycross families evaluated at Namulonge and Kachwekano during seasons 2013A and 2013B

Traits	Source						
	^a^Location, L	^b^Season, S	L*S	^c^genotype (Family)	^d^L*S*genotype (Family)	^e^Family, F	^f^Error
Storage root yield (t/ha)	7251.2**	1380.3**	2361.2*	101.3*	59.3	1297.6**	291
SPVD	48	3.1	6.6	0.9	0.5	14.4	1.8
Alternaria blight	5.2	1.3	10.5	0.9	0.5	20.7	7.4
Weevil damage	49.9**	8.3**	**73.3**	1.1**	1.1**	3.6**	1.1
Biomass (t/ha)	29814.1	20742.7	3783.4	749.2	342.9	1569.3	1078.4
Harvest index	1393.7	2799.4	19081.8	821.6	385.5	13510.8	1994.4
β-carotene content	0.6	9.0*	3.5*	3.9*	0.8	48.6**	1.5

*Note*. ** = significant at P ≤ 0.01; * = significant at P ≤ 0.05.

a Mean square tests the significant effect of location on the performance of genotypes.

b Mean squares tests the significant effects of seasons on the performance of genotypes.

c Tests the significant effect of individual genotypes (progenies derived from both polycross and controlled crosses).

d Test the significant effect of genotype × environment interaction on the on the performance of genotypes.

e Tests the significant effect of the family on the performance of progenies.

f Error term contains the interaction between crosstype with location, season and family that, plus within-trial error.

**Table 3:** Crosstype analysis of variance mean squares for seven sweetpotato traits of progenies derived by either controlled crosses or polycross evaluated at Namulonge and Kachwekano during seasons 2013A and 2013B

Traits	Source						
		^a^Location, L	^b^Season, S	L*S	^c^Crosstype	^d^L*S*crosstype	^e^Family(crosstype)	^f^Error
Storage root yield (t/ha)	7176.9**	1029.8**	3394.6**	1198.4**	827.2**	433.2**	95.6
SPVD	53.4**	6.6**	10.5**	38.2**	4.1**	2.9**	0.3
Alternaria	6.6**	1.5	11.0**	63.1**	17.7**	4.8**	3.8
Weevil damage	51.9**	12.1**	92.2**	0.2	0.1	3.7*	1
Biomass (t/ha)	25402.0**	16009.7**	7417.4**	147.7	3174.0**	971.4	1208
Harvest Index	1932.3**	5690.6**	28722.5**	14989.6**	3970.1**	48478.0**	644.7
β-carotene content	0.5	18.5*	5.9	19.0**	0.4	74.2**	2.4

*Note*. ** = significant at P ≤ 0.01; * = significant at P ≤ 0.05.

a Mean squares tests the significant effect of location on the performance of families.

b Mean squares tests the significant effects of seasons on the performance of families.

c Tests the significant effect of overall least significant mean of the cross type (polycross vs controlled cross derived progenies) on the performance of families.

d Tests the significant effect of location, season and cross type on the performance of families.

e Tests the significant effect of the parents and crossing method on the performance of families.

f Error term containss the interaction of genotype with location, season and crosstype, plus within-trial error.

Storage root yield, SPVD, Alternaria blight, biomass and HI performance of families was significantly affected by the crosstype (whether they were obtained by polycross or controlled cross method) (Table 3). The storage root yield of progenies obtained from the controlled crosses of ‘Wagabolige × NASPOT 1’ and ‘Ejumula × New Kawogo’ averaged 9.8 t/ha over the two locations and seasons while storage root yield for progenies from the polycross of ‘Wagabolige op’ and ‘Ejumula op’ was 6.5 t/ha (Table 4). The mean SPVD and Alternaria blight scores for progenies from controlled crosses over location and season was 2.1 while the mean SPVD and Alternaria blight scores for progenies from the polycross was 2.4 and 1.8, respectively. HI across locations and seasons was 43.2% for progenies derived from controlled crosses and 31.8% for progenies obtained by the polycross method.

**Table 4:** Mean performance of progenies derived from controlled crosses and polycross evaluated at Namulonge and Kachwekano during 2013A and 2013B seasons

Cross type	Storage root yield (t/ha)	SPVD	Alternaria blight	Weevil damage	Biomass (t/ha)	Harvest index	β-carotene content (mg/100 g FWB)
**Controlled**	9.8	2.1	2.1	1.8	24.5	43.2	0.4
**Polycross**	6.5	2.4	1.8	1.8	22.4	31.8	0.9
**Mean difference**	3.3	0.3	0.3	0	2.1	11.4	0.6
**LSD_(0.05)_**	0.9	0.1	0.1	0.1	2.3	2.5	0.2

**Figure 1:**
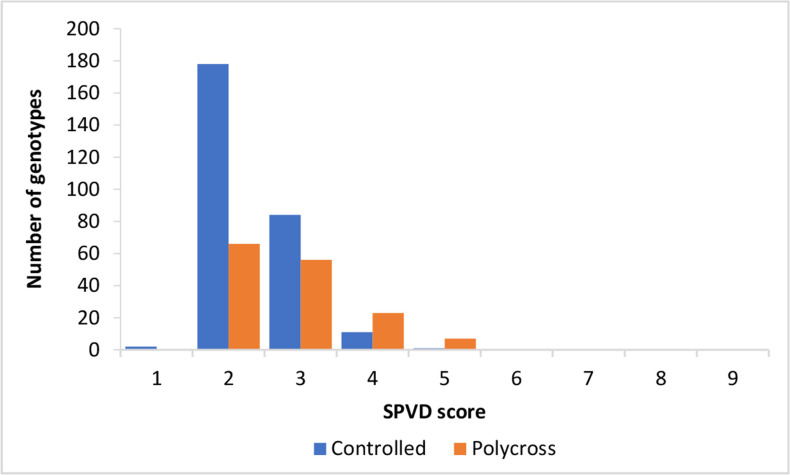
Distribution of mean sweetpotato virus disease severity in progenies derived from controlled crosses (blue) and polycrosses (orange) at Namulonge during the seasons of 2013A and 2013B

There were significant differences (P ≤ 0.05) between the polycross and controlled cross families for SPVD resistance, subsequently the controlled crosses method produced more progeny showing field resistance (SPVD score ≤ 3) (Figure 1) at the high virus pressure site of Namulonge.

**Figure 2:**
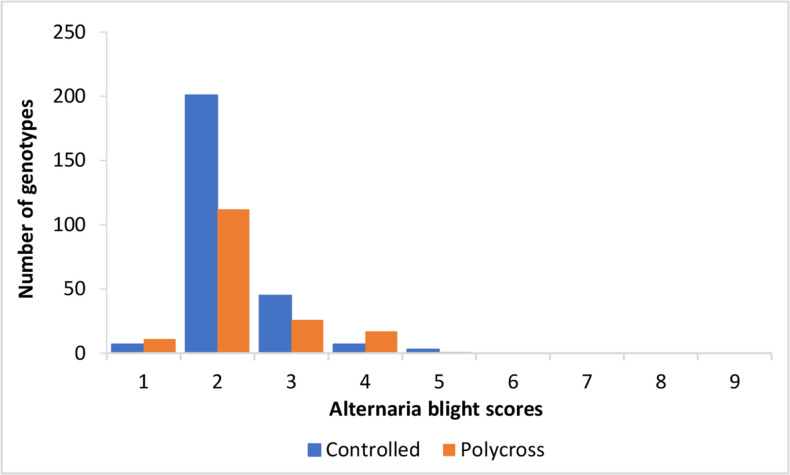
Distribution of mean Alternaria blight disease severity in progenies derived from controlled crosses (blue) and polycrosses (orange) at Kachwekano during the seasons of 2013A and 2013B

Families within cross type were significantly different (P ≤ 0.05) for Alternaria blight. The polycross families were more resistant to Alternaria blight (mean score 1.8) than the controlled cross families (mean score 2.1). However, the number of progenies showing field resistance (Alternaria blight score ≤ 3) (Figure 2) at the Alternaria blight hotspot site of Kachwekano was higher from the controlled crosses. This shows that although the polycross method was superior to the controlled cross method in generating Alternaria blight resistant genotypes, the parents used in the crossing method are very important. However, this would also require validation with more parents and larger samples.

To obtain genotypes with a good aggregate of desired traits, a SI was used. Given the different levels of importance of the traits at Namulonge and Kachwekano, separate indexes were calculated for each location. At Namulonge, the controlled crosses performed better than the polycross with 18 genotypes from controlled cross (90%) being among the top 20 genotypes with desirable traits (Table 5). However, of the 18 controlled cross genotypes, 14 genotypes (about 89%) were from ‘Wagabolige × NASPOT 1’ family. Thus, much as ‘Ejumula × New Kawogo’ and ‘Wagabolige × NASPOT 1’ were from the same crossing method, ‘Wagabolige × NASPOT 1’ produced more genotypes among the top 20 performers than ‘Ejumula × New Kawogo’. The only two polycross genotypes of the top 20 genotypes at Namulonge were from the ‘Wagabolige op’ family.

**Table 5:** Twenty top progenies based on selection index for four agronomic traits evaluated at Namulonge over two seasons in 2013

Family	Progeny (C = hand cross; P = polycross)	Storage root yield (t/ha)	Biomass (t/ha)	Harvest index (%)	SPVD	Alternaria blight	Weevil damage	β-Carotene content (mg/100 g FWB)	Selection index
Ejumula × New Kawogo	1.08C	19.0	40.0	47.5	2.5	2.5	1.0	0.0	43.9
Wagabolige op	2.49P	30.0	47.3	63.4	2.0	2.0	1.0	0.0	39.5
Wagabolige × NASPOT 1	2.163C	33.7	26.7	37.2	2.8	1.5	1.0	0.6	38.7
Wagabolige × NASPOT 1	2.57C	29.3	43.0	69.3	1.5	2.0	1.5	0.1	38.5
Wagabolige × NASPOT 1	2.135C	33.7	53.7	60.3	2.3	1.8	1.5	0.1	37.9
Wagabolige × NASPOT 1	2.70C	30.9	66.3	46.7	1.5	1.5	2.0	0.0	37.4
Wagabolige × NASPOT 1	2.75C	30.5	50.5	58.1	2.3	1.5	1.5	0.1	34.3
Wagabolige × NASPOT 1	2.08C	29.0	73.7	39.4	2.5	1.8	1.0	0.1	33.9
Wagabolige × NASPOT 1	2.19C	10.3	19.0	54.4	1.5	1.5	1.0	0.1	33.3
Wagabolige × NASPOT1	2.154C	26.0	38.0	68.4	1.8	1.5	1.5	0.0	33.1
Wagabolige × NASPOT 1	2.83C	36.4	48.7	21.6	2.3	1.8	2.5	0.1	31.6
Wagabolige × NASPOT 1	2.162C	37.3	57.3	65.3	3.0	1.5	2.0	1.4	31.2
Ejumula × New Kawogo	1.09C	27.3	45.7	54.4	2.0	2.0	1.5	0.1	30.8
Ejumula × New Kawogo	1.46C	26.5	75.1	33.8	1.5	1.8	2.0	0.1	30.5
Wagabolige × NASPOT 1	2.148C	27.4	89.4	27.2	1.8	1.5	2.0	0.0	30.1
Wagabolige × NASPOT 1	2.219C	32.3	55.0	54.9	3.0	1.5	1.5	0.8	29.4
Wagabolige × NASPOT 1	2.167C	24.9	39.9	61.3	1.5	1.8	2.0	0.1	28.3
Ejumula × New Kawogo	1.36C	13.3	26.7	50.0	2.5	2.5	1.0	0.0	28.2
Wagabolige × NASPOT 1	2.46C	21.0	29.0	71.7	2.0	1.8	1.0	0.7	27.9
Wagabolige op	2.53P	16.3	60.3	27.0	1.5	1.5	1.0	0.0	27.2

At Kachwekano, where Alternaria blight is the most important biotic constraint, the controlled cross method had 11 genotypes (55%) among the top 20 genotypes selected, of which 10 genotypes were from the ‘Wagabolige × NASPOT 1’ family (Table 6). This clearly shows that the cross combination of ‘Wagabolige × NASPOT 1’ was a superior controlled cross. This implies that for controlled crosses, selecting the best combination of the parents is very valuable. However, when the selections are made from both locations (Namulonge and Kachwekano), 15 genotypes (75%) of top 20 genotypes are from the controlled crosses (Table 7). However, the best three genotypes (15%) with a good aggregate of the desired traits are from the polycross family of ‘Ejumula (op)’ (Table 7).

**Table 6:** Twenty top progenies based on selection index for four agronomic traits evaluated at Kachwekano over two seasons in 2013

Family	Progeny (C = hand cross; P = polycross)	Storage root yield (t/ha)	Biomass (t/ha)	Harvest Index (%)	SPVD	Alternaria blight	Weevil damage	β-Carotene content	Selection index
Ejumula op	1.70P	57.6	59.2	97.3	1.0	1.5	1.0	4.0	87.9
Ejumula op	1.67P	47.7	48.9	97.5	2.5	1.5	1.0	1.4	64.3
Ejumula op	1.68P	43.6	44.8	97.3	2.0	1.5	1.0	0.1	61.9
Wagabolige × NASPOT 1	2.150C	53.9	100.5	53.6	1.5	1.5	4.0	0.1	49.4
Wagabolige × NASPOT 1	2.08C	28.4	109.7	25.9	2.0	1.0	1.0	0.2	46.0
Wagabolige × NASPOT 1	2.135C	29.2	62.6	50.8	2.0	1.3	1.0	0.1	44.6
Wagabolige × NASPOT 1	2.126C	12.8	50.5	25.4	3.3	1.5	1.0	0.0	40.4
Ejumula op	1.66P	31.6	35.2	82.2	1.5	2.3	1.5	1.2	36.2
Wagabolige × NASPOT 1	2.163C	21.2	45.2	46.9	1.5	1.5	1.0	0.0	34.4
Wagabolige × NASPOT 1	2.100C	23.6	46.9	50.3	2.0	1.5	1.0	6.1	34.3
Ejumula op	1.62P	17.1	17.9	69.2	1.5	1.3	1.0	2.1	31.2
Wagabolige × NASPOT 1	2.01C	17.3	56.7	30.6	1.5	1.5	1.0	1.5	29.0
Wagabolige op	2.35P	17.5	20.3	60.0	1.8	1.0	1.5	0.1	27.5
Wagabolige × NASPOT 1	2.174C	21.0	38.3	45.9	1.5	1.8	1.5	0.1	26.6
Wagabolige × NASPOT 1	2.180C	33.3	60.0	55.6	1.5	2.0	3.0	0.0	26.1
Wagabolige op	2.42P	16.1	56.5	28.6	1.8	1.0	1.5	0.0	25.7
Ejumula × New Kawogo	1.40C	17.1	32.1	53.1	2.0	1.5	1.0	0.0	25.3
Ejumula op	1.57P	19.5	32.1	60.6	2.5	1.5	1.0	0.1	25.3
Wagabolige op	2.30P	18.0	36.7	40.7	1.5	1.5	1.5	0.0	24.9
Wagabolige × NASPOT 1	2.136C	16.9	67.5	30.3	1.5	1.5	1.5	0.1	23.4

**Table 7:** Top twenty progenies at both Namulonge and Kachwekano based on selection index for four agronomic traits evaluated over two seasons in 2013

Family	Progeny (C = handcross; P = polycross)	Storage root yield (t/ha)	Biomass (t/ha)	Harvest Index (%)	SPVD	Alternaria blight	Weevil damage	Carotene content	Selection Index
Ejumula op	1.70P	57.6	59.2	97.3	1.0	1.5	1.0	4.0	87.9
Ejumula op	1.67P	47.7	48.9	97.5	2.5	1.5	1.0	1.4	64.3
Ejumula op	1.68P	43.6	44.8	97.3	2.0	1.5	1.0	0.1	61.9
Wagabolige × NASPOT 1	2.150C	53.9	100.5	53.6	1.5	1.5	4.0	0.1	49.4
Wagabolige × NASPOT 1	2.08C	28.4	109.7	25.9	2.0	1.0	1.0	0.2	46.0
Wagabolige × NASPOT 1	2.135C	29.2	62.6	50.8	2.0	1.3	1.0	0.1	44.6
Ejumula × New Kawogo	1.08C	19.0	40.0	47.5	2.5	2.5	1.0	0.0	43.9
Wagabolige × NASPOT 1	2.126C	12.8	50.5	25.4	3.3	1.5	1.0	0.0	40.4
Wagabolige op	2.49P	30.0	47.3	63.4	2.0	2.0	1.0	0.0	39.5
Wagabolige × NASPOT 1	2.163C	33.7	26.7	37.2	2.8	1.5	1.0	0.6	38.7
Wagabolige × NASPOT 1	2.57C	29.3	43.0	69.3	1.5	2.0	1.5	0.1	38.5
Wagabolige × NASPOT 1	2.135C	33.7	53.7	60.3	2.3	1.8	1.5	0.1	37.9
Wagabolige × NASPOT 1	2.70C	30.9	66.3	46.7	1.5	1.5	2.0	0.0	37.4
Ejumula op	1.66P	31.6	35.2	82.2	1.5	2.3	1.5	1.2	36.2
Wagabolige × NASPOT 1	2.163C	21.2	45.2	46.9	1.5	1.5	1.0	0.0	34.4
Wagabolige × NASPOT 1	2.100C	23.6	46.9	50.3	2.0	1.5	1.0	6.1	34.3
Wagabolige × NASPOT 1	2.75C	30.5	50.5	58.1	2.3	1.5	1.5	0.1	34.3
Wagabolige × NASPOT 1	2.08C	29.0	73.7	39.4	2.5	1.8	1.0	0.1	33.9
Wagabolige × NASPOT 1	2.19C	10.3	19.0	54.4	1.5	1.5	1.0	0.1	33.3
Wagabolige × NASPOT 1	2.154C	26.0	38.0	68.4	1.8	1.5	1.5	0.0	33.1

Broad-sense heritability (h^2^) of observed quantitative traits was calculated as a ratio of the genetic variance and the phenotypic variance components. The broad sense heritability for storage root yield, biomass, HI and β-carotene content varied from 30% (storage root yield) to 100 (β-carotene content and HI) (Table 8). The variance component estimates of the interaction of the genotypes with the environment ($\delta_{GxLxS}^{2}$) was far greater than the genetic variance ($\delta_{G}^{2}$) estimates for storage root yield. This implies that unlike other quantitative traits like biomass, HI and β-carotene content, sweetpotato breeding efforts for storage root yield will be affected by the strong G×E interactions and progenies ought to be analyzed for yield stability.

**Table 8:** Variance component estimations for the trials conducted at Namulonge and Kachwekano to compare the performance of genotypes generated by polycross and controlled crosses for two the seasons of 2013

Trait	$\delta_{L}^{2}$	$\delta_{S}^{2}$	$\delta_{GxLxS}^{2}$	$\delta_{G}^{2}$	$\delta_{\text{err }}^{2}$	$\delta_{P}^{2}$	H^2^
Storage root yield (t^2^/ha^2^)	16.1	4.6	65.0	17.1	0.7	54.0	30.0
Biomass (t^2^/ha^2^)	54.4	29.3	0.0	144.2	350.0	228.1	60.0
HI	1.6	0.4	0.0	168.0	447.8	170.1	98.8
β-Carotene content	0.0	0.0	0.9	1.3	0.0	1.3	100.0

## 4. Discussion

This study was conducted to compare the two sweetpotato breeding methods: the controlled cross method and the polycross method. This information will be useful in improving the efficiency of breeding programs. Significance of the cross type (polycross or controlled cross) for storage root yield, SPVD, Alternaria blight and HI indicates that the crossing method is very important in generating genotypes with resistance to SPVD and Alternaria blight and improving storage root yield and HI. For instance, there were significant differences (P≤0.05) between the polycross and controlled cross families for SPVD resistance, subsequently the controlled crosses method produced more progeny showing field resistance (SPVD score ≤ 3) (Figure 1) at the high virus pressure site of Namulonge. This indicates that the crossing method is very important in generating SPVD resistant progenies. Considerable genetic variation for SPVD resistance has been reported in sweetpotato breeding populations (Gruneberg et al., 2015). It is plausible to attribute the superiority of the controlled cross method to the very low frequencies (≤ 0.2%) of high resistance levels to SPVD in breeding populations which, necessitates carefully selecting parents (Mwanga et al., 2002). Strategies for selecting parents can be based on the performance of parents per se, the performance of their progenies or molecular markers in genomic selection models or a combination of the different strategies (Witcombe & Virk, 2009; Burgueño et al., 2012). Unfortunately, the study did not consider inclusion of parents to allow for parent-offspring analysis. Similar studies in future will be able to deploy DNA markers for SPVD resistance that are currently in the experimental validation phase and genomic selection tools for sweetpotato are under development (Mwanga et al., 2017; Wu et al., 2018).

Improvement for storage root yield is high priority in sweetpotato breeding programmes all over the world. Higher storage root yield can be achieved by either increasing the total biological yield (biomass) or increasing the proportion of the biomass allocated to storage roots, thus increasing the HI (Gruneberg et al 2015). This study shows that the crossing method does not affect significantly (P ≤ 0.05) the expression of biomass. Biomass is an indicator of the plant’s net assimilation ability and our results show that both crossing methods can be used to successfully improve it. However, the crossing method significantly (P ≤ 0.05) affected the allocation of biomass into storage root yield, subsequently affecting the HI among the families. The controlled cross families had a significantly higher mean HI, 43.2% than the polycross families with a mean HI of 31.8% (Table 4). This indicates that larger genetic gains for storage root yield could be obtained by increasing the HI from controlled crosses than the polycross method. Therefore, controlled crosses can be deployed to systematically increase the HI in breeding populations. The significant effect of family within cross type on both storage root yield and HI continue to emphasize the importance of the attributes of the parents used in the crossing method.

The significance (P ≤ 0.05) of the differences between the four families within cross type for all traits indicate that the families reacted differently for the different traits. Further highlighting that the parents used in a cross are very important in generating genotypes with desired attributes. This underscores the need to for breeding sweetpotato programs to invest in pre-breeding studies to select parents to be used in either controlled crosses or a polycross that will produce acceptable, high yielding, pest and disease resistant progenies. For instance, understanding the combining abilities, mid-parental values and divergence coefficients among the parents before they are used in either a controlled cross or in a polycross is crucial. For example, in the selection indices, most of the genotypes that constituted the top 20 performers at the two sites from the controlled crosses came from ‘Wagabolige’ × ‘NASPOT 1’ cross and very few from ‘Ejumula’ × ‘New Kawogo’. However, the best three genotypes (15%) with a good aggregate of the desired traits are from the polycross family of ‘Ejumula (op)’ (Table 7). This shows that unlike the biparental controlled cross method, the polycross method which is generating progenies from a wider genetic background provides sufficient variability to allow selection of varieties combining many desirable attributes. Most breeding programs in SSA have made selections for variety release from the polycrosses (Gruneberg et al., 2015). This study shows that controlled crosses also generate many top performing genotypes indicating the potential of the method to increase the frequency of high performing genotypes and could be very useful for population improvement. It was apparent that both the polycross and controlled crosses are good methods for generating new sweetpotato genotypes in a sweetpotato breeding program. In subsequent studies, a comparison of the cost of implementing a breeding program using either of the two crossing methods in view of the genetic gain realised may be beneficial.
